# Editorial: Social identity dynamics in a networked society

**DOI:** 10.3389/fpsyg.2023.1264534

**Published:** 2023-08-10

**Authors:** Joseph Hafer, Bing Ran, P. Robert Duimering

**Affiliations:** ^1^Public and Nonprofit Administration, University of Memphis, Memphis, TN, United States; ^2^School of Public Affairs, Penn State Harrisburg, Middletown, PA, United States; ^3^Management Science and Engineering, University of Waterloo, Waterloo, ON, Canada

**Keywords:** social identity, information, algorithmized information, information processing, networked society

The study of social identity has been at the forefront of social psychology and behavioral research for decades. Social identity theory and self-categorization theory emerged as approaches to conceptualize how the self-concept relates to identities in social contexts and how these identities impact intergroup dynamics and information processing (Hornsey, 2008; Ramarajan, 2014). However, the role of social identity in an increasingly networked society is not well understood. The ubiquitous flow of algorithmized information impacts identity, behavior, and cognition in complex ways (Lee et al., 2022). Social media and other information and communications technology (ICT) organizations use personalization algorithms to model a person's identity, which then influences the kinds of information shared with the person, and the options and resources presented to them in different decision situations (e.g., ads/offers targeted for certain demographics). Thus, social category membership can influence an individual's knowledge, attitudes, and beliefs, which in turn affects how they perceive and interpret events, make decisions, and collaborate with others to solve problems. Such effects have been linked to increased polarization in society (Van Bavel et al., 2021), but little is known about the underlying psychological and cognitive processes involved.

This special Research Topic within *Frontiers in Psychology* on “Social identity dynamics in a networked society” has curated eight articles that begin to address the knowledge gap noted above in three ways. First, three articles explore behavioral consequences of the links between social identity and the hyper-networked society. The findings of Li et al. suggest that when people perceive inconsistency in others on social media (e.g., online vs. offline behavior), the interpersonal evaluations of those perceiving the inconsistency are impacted by the social identities they hold as they use that information to judge the authenticity and motives of the other on social media. Analyzing different crowd behaviors that shifted during the COVID-19 pandemic (e.g., panic buying) through case analysis and simulations, Brindal et al. find that social identity theory and self-schema theory help to explain the ingroup favoritism and outgroup bias that resulted from individual decisions and behavior and led to collective outcomes for crowd behaviors. Lastly, Manago et al. highlight the role of personal identification with perceived social identities in shaping individual differences in social media use, and demonstrate how gender identification, traditional masculinity and femininity ideologies contribute to the purposes of social media use during adolescence (such as how femininity ideology is associated with using social media for emotional bonding, while masculinity ideology is linked to using social media for competitive activity bonding).

Second, three articles highlight reinforcing cycles of social media use and social identity formation. Yu finds that online social networking by university students can produce positive social learning outcomes, such as “whole person” development via explicit self-conceptualization, and suggests that there is a need to address the gap between viewing social media as “learning space” vs. “personal space”, particularly in a university (or academic) setting. Using serial mediation regression models, Chang et al. found a mediating link between social identity formation and “aggressive participation” in social media behavior such that, “when users are immersed in social media use (unconscious), they will identify with the community leading to a higher path coefficient of aggressive participation than that of community identification (conscious)”. Through structural equation modeling and path analysis, Gu et al. demonstrate that self-identity formation is linked to behavior in online knowledge communities (such as novel posting) and that such behavior is positively affected by the social identity linked to those communities, highlighting the reciprocal relationship between different levels of identity and the online, networked society.

Third, two articles look at personal outcomes of the networked digital world. Karakose et al. use bibliometric and science mapping analysis to demonstrate the importance of understanding the reciprocal relationship between digital addiction (such as social media use) and depression, particularly for adolescents and young adults. Research that focuses on preventive strategies is lacking. Lastly, Tao and Scott investigate the impact of online discrimination on African American adolescents, revealing that such experiences can lead to negative outcomes and internalized stereotypes. It also explores how vicarious online discrimination, parental technological attitudes, and racial identity centrality influence these experiences, highlighting the complex interplay of social identities in a networked society.

The articles in this Research Topic only scratch the surface for understanding the dynamic interplay between social identity and accessibility of information and personal connections across the globe. The utilization of diverse methodological approaches highlights the need to question what is the best data to understand the dynamic interplay and who has access to this data (is it limited to ICT organizations)? What level and form of regulatory oversight or government intervention is appropriate? In addition, while biased information has always been available, its omnipresence has made it exceedingly easy for people to live within their own echo-chambers. Once individuals become deeply entrenched in this confinement of biased information, are there successful interventions that can pull them out? Can those interventions be scaled to a societal level? And at the core of the issue - how do individuals reconcile underlying internal cognitive motivations and conflict (e.g., cognitive dissonance) during social identity construction, alignment, or realignment and how do social media policy interventions influence this process? The editors of this Research Topic look forward to future research in this area and the potential impact it can make for positive societal change.

## Author contributions

JH: Writing—original draft, Writing—review and editing. BR: Writing—original draft, Writing—review and editing. PD: Writing–original draft, Writing—review and editing.
